# The prognostic capacities of CBP and p300 in locally advanced rectal cancer

**DOI:** 10.1186/s12957-019-1764-8

**Published:** 2019-12-19

**Authors:** Felix Rühlmann, Indra Maria Windhof-Jaidhauser, Cornelius Menze, Tim Beißbarth, Hanibal Bohnenberger, Michael Ghadimi, Sebastian Dango

**Affiliations:** 1 Department of General, Visceral and Pediatric Surgery, University Medical Center, Robert-Koch-Str. 40, 37075 Göttingen, Germany; 2 Department of Medical Statistics, University Medical Center, Göttingen, Germany; 3 Department of Pathology, University Medical Center, Göttingen, Germany; 4 Department of General and Visceral Surgery, Kreisklinikum Siegen, Weidenauer Str. 76, 57076 Siegen, Germany

**Keywords:** Colorectal cancer, Biomarkers, Targeted therapy, Histone acetyltransferase

## Abstract

**Background:**

CREB-binding protein (CBP) and p300 represent histone acetyltransferases (HATs) and transcriptional coactivators that play essential roles in tumour initiation and progression. Both proteins are generally thought to function as tumour suppressors, although their distinct roles in colorectal cancer (CRC) remain inconsistent and ambiguous.

Thus, we analysed the expression of these two HATs in human tissue samples from patients with locally advanced rectal cancer via immunohistochemistry and evaluated their potential impacts on future CRC diagnosis and treatment.

**Methods:**

In our analysis, we included ninety-three (*n* = 93) patients diagnosed with adenocarcinoma in the upper third of the rectum. None of the patients received preoperative chemoradiotherapy, but the patients did undergo primary resection of the tumour within the phase II GAST-05 trial. By using H-scores, the expression of both proteins was visualised via immunohistochemistry in resected specimens from the patients. CBP and p300 expression were correlated with clinical and follow-up data.

**Results:**

Our analysis showed that high expression of CBP was significantly associated with prolonged cancer-specific survival (CSS; *p* = 0.002). In univariate analysis, CBP was an independent prognostic parameter for CSS (*p* = 0.042). High nuclear CBP expression was observed in two-thirds of patients. In contrast, we could not find any significant correlation between the expression of p300 and cancer-specific survival in this cohort of patients (*p* = 0.09). We did not observe any cooperation between CBP and p300 in our analysis.

**Conclusions:**

High expression of CBP was significantly associated with improved oncological outcomes. This finding could help to stratify patients in the future for CRC treatment. Histone deacetylase (HDAC) inhibitors are increasingly playing a role in oncological treatment and could additionally become therapeutic options in CRC. Our findings need to be further evaluated and verified in future clinical analyses.

## Background

Colorectal cancer (CRC) represents one of the most common malignancies in the Western world [1]. Although advances in perioperative radiation and chemotherapy have been made, including the implementation of specific monoclonal antibodies, the long-term prognosis of CRC is still limited because patients respond heterogeneously to current standard treatments. Therefore, individualised therapies are desirable. Thus, it is essential to find potential biomarkers and new therapeutic targets to improve patient outcomes.

As epigenetic alterations such as DNA methylation and histone modifications have raised interest regarding the initiation and progression of tumours in recent decades, epigenetic therapies addressing epigenetic modifiers are now being included in clinical trials for cancer treatment [2, 3]. The highly conserved tumour suppressor and transcriptional coactivator CREB binding protein (CBP) as well as its close partner p300 are histone acetyltransferases (HATs) that share approximately 60% homology and play essential roles in gene expression regulation by acetylating chromatin substrates [4, 5].

The acetylation of histones leads to a reduction in the electrostatic interactions between the positive charge of histones and the negative charge of DNA, which reduces the compactness of the chromatin structure and favours transcriptional progression, possibly contributing to carcinogenesis by specific activation of cancer-associated genes [6].

Studies suggest an inverse correlation between the expression of CBP and that of p300 because these molecules correlate positively and negatively with patient survival. Despite the high degree of homology between these proteins, studies have revealed functional differences from other HAT proteins due to differences in substrate specificities [7]. CBP and p300 are involved in several cellular activities such as cell growth, differentiation, DNA repair and apoptosis. They also interact with at least 40 different transcription factors [7–9]. Previous studies have shown that the interaction between CBP/p300 and ß-Catenin influences Wnt/ß-Catenin signalling. The influence of CBP and p300 activity on Wnt/ß-Catenin signalling affects cell proliferation and differentiation [10–14]. Mutations in this pathway are responsible for the initiation of many CRC tumours [15].

Somatic alterations in the CBP gene are associated with malignant diseases such as acute myeloid leukaemia and hepatocellular carcinoma, while germline mutations in the *CBP* gene have been identified in Rubinstein-Taybi disease [16–18]. Mutations in p300 have recently been detected in colon cancer and gastric cancer [19].

Although dysfunction in CBP and/or p300 is considered to be associated with tumourigenesis in several human malignancies, their roles in CRC remain unclear and somewhat controversial. Therefore, we investigated the expression of CBP and p300 in patients with rectal adenocarcinoma via immunohistochemistry, and the findings were compared with clinicopathological parameters, including patient outcome, to investigate the clinical impacts and functions of both the tumour suppressor CBP and the potential oncogene p300. In addition, molecular aspects in the context of potential downstream targets were analysed. Herein, we show for the first time that CBP overexpression in CRC but not p300 overexpression is associated with an improved outcome.

## Methods

### Patients

Specimens from patients with locally advanced UICC (Union International Contre le Cancer) II/III colorectal adenocarcinoma in the upper third of the rectum included in the phase II GAST-05 trial were assessed using immunohistochemistry. Study details of the GAST-05 trial are described elsewhere [20].

Patients with complete follow-up were further analysed. Approval from the local ethics committee and informed consent from patients were given (study number 9/8/08). Written consent was obtained from all 93 patients.

Patients were treated at the Department of General, Visceral and Paediatric Surgery, University Medical Center Göttingen (UMG), Germany, between March 2007 and September 2012.

### Histopathological assessment

Histopathological and clinical staging included TNM staging as well as grading and tumour stage classification [21]. Nodal staging was evaluated histopathologically by examining all detected lymph nodes and determining the lymph node ratio in all cases. Complete lymph node dissection data were included once 12 or more lymph nodes were found in the resected tissue and were taken for further analysis as recommended. Tumour tissue was collected at the time of surgery.

### Immunohistochemical determination of CBP/p300 statuses

CBP and p300 expression were assessed using formalin-fixed, paraffin-embedded (FFPE) tissue samples from resection specimens cut into sections with a thickness of 2 μm. Standardised immunohistochemical staining was performed using a polyclonal rabbit anti-CBP antibody (Catalogue No. IHC-00023, Bethyl, Montgomery, TX, USA, 1:50 dilution). Heat-mediated epitope retrieval was performed for 90 min at 100 °C. The anti-CBP antibody was incubated at room temperature for 30 min. Staining was visualised by means of alkaline phosphatase using the ultraView Universal Fast Red Kit (Ventana Medical Systems).

**Fig. 1:**
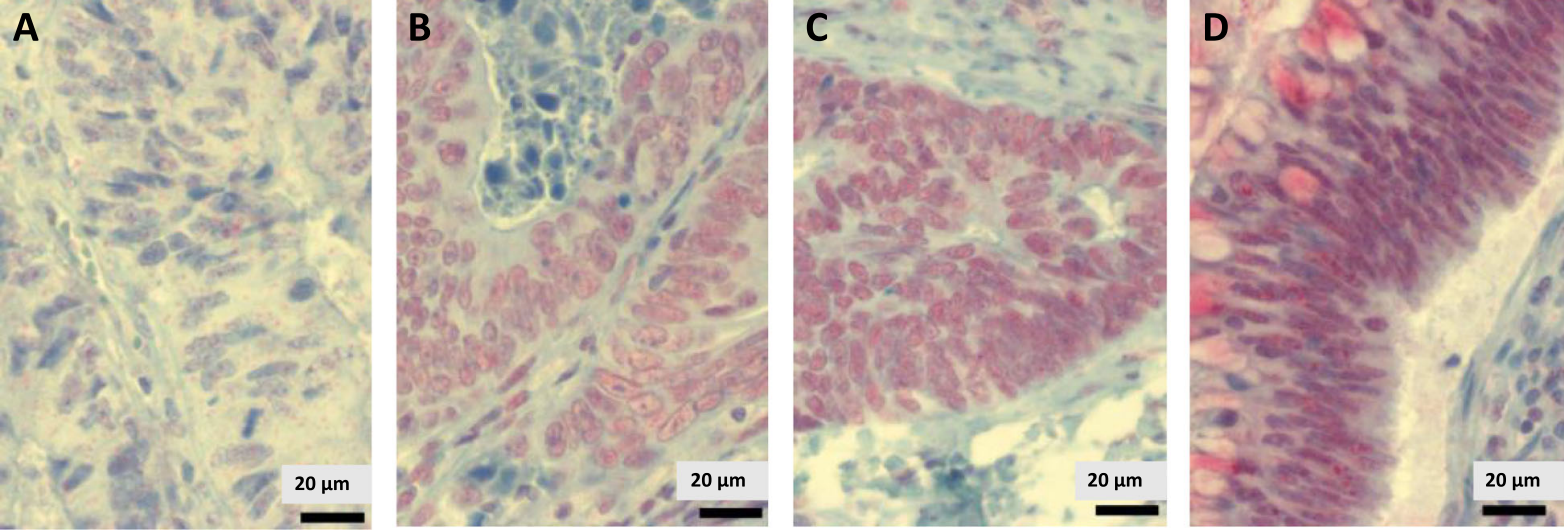
Immunohistochemical staining for CBP expression in CRC cells. **a** Very weak CBP staining (intensity 0). **b** Weak CBP staining (intensity I), **c** Strong CBP staining (II) **d** Very strong CBP staining (III)

The monoclonal mouse *anti-p300 antibody ab3164* (Abcam, Cambridge, Great Britain, 1:500 dilution) was incubated at 37 °C for 40 min. For p300, heat-mediated epitope retrieval was performed for 56 min at 100 °C. Horseradish peroxidase was used for visualisation, and staining was analysed using the optiView Universal DAB Detection Kit (Ventana Medical Systems) (Fig. 1).

**Fig. 2:**
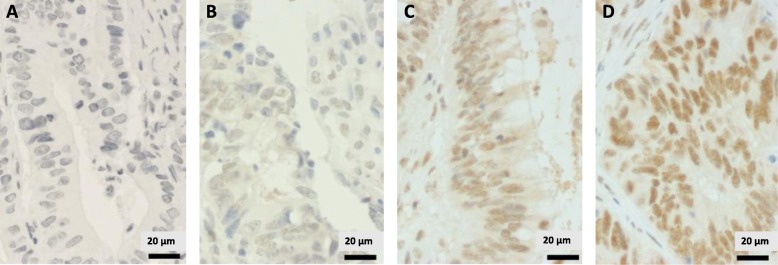
Immunohistochemical staining for p300 expression in CRC cells. **a** Very weak p300 staining (intensity 0). **b** Weak p300 staining (intensity I), **c** Strong p300 staining (II). **d** Very strong p300 staining (III)

Standard immunohistochemical staining was performed on a Ventana Bench-Mark XT immunostainer (Ventana, Tucson, AZ, USA). More than 100 tumour cells were needed in resection specimens to define CBP and p300 positivity. Since both CBP and p300 are located in the nucleus, nuclear staining was exclusively analysed. In order to quantify immunohistochemical staining, H-score was implemented as described before ranging from 0 to 300 (*y*-axis). For nuclear staining, four different staining intensity grades were defined: 0 (very weak staining), I (weak staining), II (strong staining) and III (very strong staining). For every immunohistochemical slide, we chose three different areas, which were localised (a) at the basis of the tumour on the boundary layer to healthy tissue, (b) at the centre of the tumour and (c) at the apex towards the gut lumen. This area approximately covers 7.500 μm^2^ (Fig. 2).

### Statistical analysis

The expression of CBP and p300 was correlated with clinicopathological parameters as described. The impacts of CBP and p300 on disease-free survival (DFS) and cancer-specific survival (CSS) were determined using Kaplan-Meier analysis and assessed for statistical significance using Kendall’s Tau (Cox proportional hazard model). Statistical analysis was performed using the R package (version 2.14.2), and survival analysis was carried out after grouping patients into three groups (high, medium, and low) as described above [22]. The significance level was set at *α* = 5%. To quantify immunohistochemical staining, the H-score was implemented as described previously [23].

## Results

### Patient characteristics and recurrence

All analysed patients were registered in the prospective multi-centre phase II GAST-05 trial. Overall, 93 patients with a confirmed adenocarcinoma in the upper third of the rectum underwent surgery, partially followed by postoperative chemotherapy. In this analysis, 63 patients (67.7%) were male, while 30 patients were female (32.3%). Patient ages ranged from 38 to 88 years (median 70 years). In this analysis, anterior rectal resection (ARR) was performed in 37 patients (39.8%), while 53 patients (57.0%) underwent lower anterior rectal resection (LARR). In three patients (3.2%), Hartmann’s procedure was performed. Regarding the extent of mesorectal excision, total mesorectal excision (TME) was executed in 38 patients (40.9%), while 52 patients (55.9%) experienced a partial mesorectal excision (PME).

Within this cohort of patients, 3 patients (3.2%) presented with early pT1-stage disease, while 18 patients (19.4%) were diagnosed with pT2 tumours. Within the patients with locally advanced rectal tumours, 61 patients (65.6%) had pT3 tumours, while in 11 patients (11.8%), a pT4 tumour was present. Considering the lymph node status of the patients, 53 patients (57%) did not show any lymph node metastasis, while 22 patients (23.7%; pN1) and 18 patients (19.3%; pN2) were diagnosed with positive lymph nodes. Regarding long-term follow-up, 3 patients (3.2%) developed local recurrences, while 15 patients (16.1%) showed distant metastases (for details, see Table 1).

**Table 1:** Correlations between CBP/p300 expression and clinicopathological parameters

Variable	No. of patients	CBP expression			p300 expression			*p* value
		Low	Medium	High	Low	Medium	High	
Total	93	28 (30.1%)	33 (35.5%)	32 (34.4%)	31 (33.3%)	30 (32.3%)	32 (34.4%)	0.86
Female	30	7 (23.3%)	11 (36.7%)	12 (40.0%)	7 (23.3%)	10 (33.3%)	13 (43.3%)	0.96
Male	63	21 (33.3%)	22 (34.9%)	20 (31.7%)	24 (38.1%)	20 (31.7%)	19 (30.2%)	0.85
Age (years)								
< 60	68	25 (36.8%)	21 (30.9%)	22 (32.4%)	25 (36.8%)	19 (27.9%)	24 (35.3%)	0.91
≥ 60	25	3 (12.0%)	12 (48.0%)	10 (40.0%)	6 (24.0%)	11 (44.0%)	8 (32.0%)	0.53
Tumour infiltration								
pT1	3	2 (66.7%)	0 (0%)	1 (33.3%)	0 (0%)	1 (33.3%)	2 (66.6%)	0.19
pT2	18	6 (33.3%)	5 (27.8%)	7 (38.9%)	4 (22.2%)	6 (33.3%)	8 (44.4%)	0.76
pT3	61	18 (29.5%)	23 (37.7%)	20 (32.8%)	23 (37.7%)	22 (36.1%)	16 (26.2%)	0.58
pT4	11	2 (18.2%)	5 (45.5%)	4 (36.3%)	4 (36.4%)	1 (9.1%)	6 (54.5%)	0.15
Lymph node status								
pN0	53	15 (28.3%)	18 (34.0%)	20 (37.7%)	16 (30.2%)	18 (34.0%)	19 (35.8%)	0.97
pN1	22	7 (31.8%)	9 (40.9%)	6 (27.3%)	8 (36.4%)	8 (36.4%)	6 (27.3%)	0.94
pN2	18	6 (33.3%)	6 (33.3%)	6 (33.3%)	7 (38.9%)	4 (22.2%)	7 (38.9%)	0.76
Lymphatic vessel invasion								
L0	68	20 (29.4%)	24 (35.3%)	24 (35.3%)	20 (29.4%)	24 (35.3%)	24 (35.3%)	1
L1	23	7 (30.4%)	9 (39.1%)	7 (30.4%)	10 (43.5%)	6 (26.1%)	7 (30.4%)	0.57
LX	2	1 (50.0%)	0 (0%)	1 (50.0%)	1 (50.0%)	0 (0%)	1 (50.0%)	n/a
Vein invasion								
V0	80	24 (30.0%)	27 (33.8%)	29 (36.2%)	26 (32.5%)	25 (31.2%)	29 (36.2%)	0.92
V1	12	4 (33.3%)	6 (50.0%)	2 (16.7%)	5 (41,7%)	5 (41.7%)	2 (16.7%)	0.9
VX	1	0 (0%)	0 (0%)	1 (100.0%)	0 (0%)	0 (0%)	1 (100%)	n/a
Grade								
G1	0	0 (0%)	0 (0%)	0 (0%)	0 (0%)	0 (0%)	0 (0%)	n/a
G2	67	20 (30.0%)	25 (37.2%)	22 (32.8%)	23 (34.3%)	21 (31.3%)	23 (34.3%)	0.75
G3	25	8 (32.0%)	8 (32.0%)	9 (36.0%)	8 (32.0%)	9 (36.0%)	8 (32.0%)	0.94
GX	1	0 (0%)	0 (0%)	1 (100%)	0 (0%)	0 (0%)	1 (100.0%)	n/a
Resection boundaries								
R0	89	28 (31.5%)	31 (34.8%)	30 (33.7%)	30 (33.7%)	30 (33.7%)	29 (32.6%)	0.95
R1	4	0 (0%)	2 (50.0%)	2 (50.0%)	1 (25.0%)	0 (0%)	3 (75.0%)	0.2
UICC								
pUICC I	14	6 (42.9%)	1 (7.1%)	7 (50.0%)	3 (21.4%)	5 (35.7%)	6 (42.9%)	0.15
pUICC II	39	9 (23.0%)	17 (43.7%)	13 (33.3%)	13 (33.3%)	13 (33.3%)	13 (33.3%)	0.53
pUICC III	38	13 (34.2%)	14 (36.9%)	11 (28.9%)	15 (39.5%)	12 (31.6%)	11 (28.9%)	0.86
pUICC IV	2	0 (0%)	1 (50.0%)	1 (50.0%)	0 (0%)	0 (0%)	2 (100.0%)	n/a

The expression patterns of both enzymes were grouped according to the expression intensity as indicated below. *pT* histological tumour size, *pN* histological lymph node status, *L* invasion in lymphatic vessels, *V* invasion in venous vessels, *G* grade, *R* resection boundaries, and *pUICC* (Union Internationale Contre le Cancer) histological classification for malignant tumours. *p* values were determined using the chi-squared test

### CBP expression in resection specimens evaluated by immunohistochemistry

**Fig. 3:**
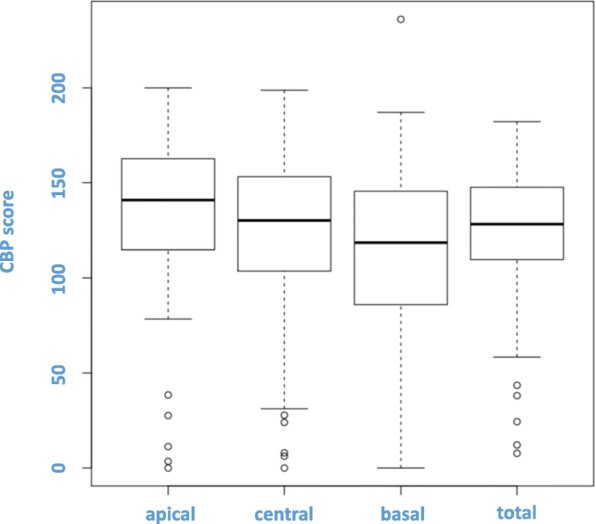
Immunohistochemical expression of CBP. No correlations were found between protein expression and localization within the nucleus

**Fig. 4:**
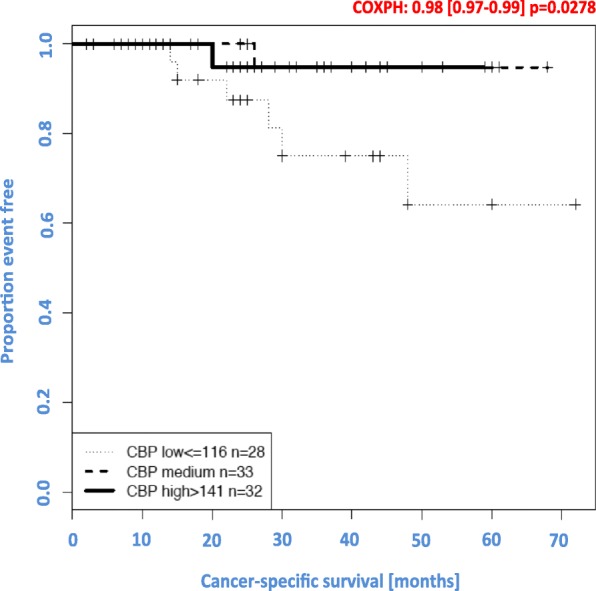
Correlation between the expression of CBP and CCS. High expression of CBP was significantly associated with prolonged cancer-specific survival (*p* = 0.002)

CBP expression was exclusively nuclear, and no significant correlation was observed between CBP expression and apical, central or basal localisation of CBP (see Fig. 3). High expression of CBP was significantly associated with prolonged CSS (*p* = 0.002; see Fig. 4). Furthermore, subgroup analysis showed a correlation between high and medium CBP expression (*p* < 0.05), but not between low expression and CSS (*p* > 0.05). In this cohort of patients, CBP expression represented an independent prognostic parameter for CSS by univariate analysis (*p* = 0.042). There were no further significant associations between CBP expression and clinicopathological parameters, e.g. size of the tumour (pT), postoperative nodal status (pN), distant metastasis status, pUICC or grade. Additionally, there was no significant correlation between the expression of CBP and local relapse (*p* > 0.05).

### p300 expression in resection specimens evaluated by immunohistochemistry

**Fig. 5:**
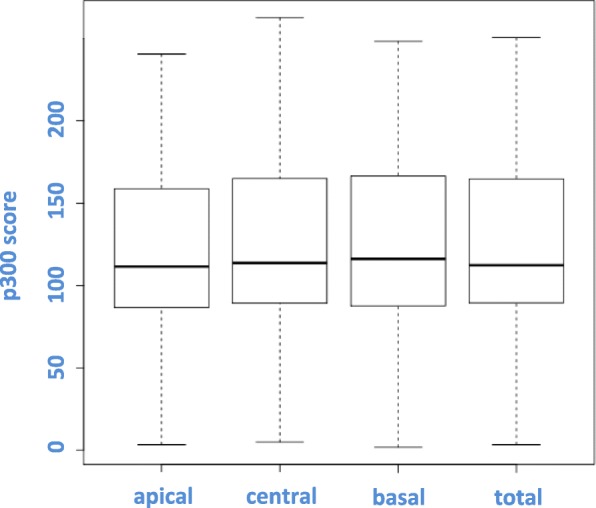
Distribution of nuclear p300 expression. Correlations between the localization of CBP within the nucleus and the intensity of CBP expression

**Fig. 6:**
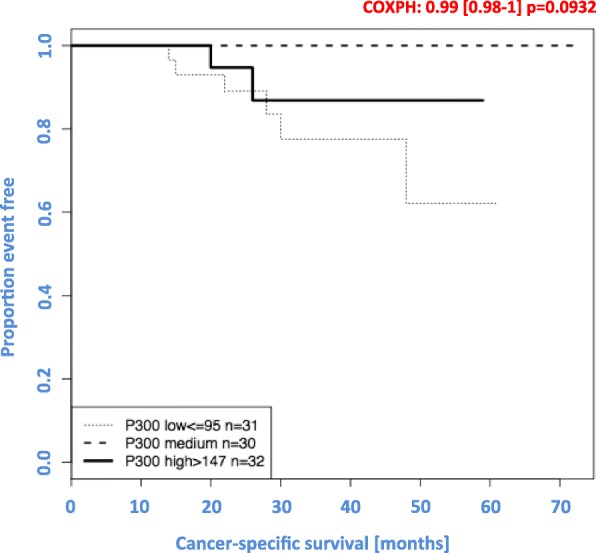
Correlation between the expression of p300 and CCS. Low expression of p300 was non-significantly correlated with poor cancer-specific survival (*p* = 0.093)

The expression of p300 was exclusively nuclear. There was no predominant localization in the apical, central or basal side (see Fig. 5). Low expression of p300 was not significantly associated with poor CSS (*p* = 0.09; see Fig. 6). In our hands, p300 did not represent a prognostic parameter. We could not find any correlations between p300 expression and clinicopathological parameters such as tumour size (pT), lymph node status (pN), distant metastasis status, pUICC or grade. Furthermore, we could not find any association between p300 expression and local relapse.

## Discussion

CBP and p300 function as transcriptional coactivators and HATs, which may favour euchromatin formation and therefore activate transcriptional activity. Studies have shown that both enzymes are involved in numerous cellular activities such as cell growth, differentiation, DNA repair and apoptosis. However, their distinct roles in CRC remain unclear.

Previous studies suggested an inverse correlation between the expression of CBP and p300 in CRC with regard to overall survival (OS) [24]. Correlations have also been observed in other cancers, such as prostate cancer, where genetic deletion of CBP and p300 results in the promotion or perturbation of tumourigenesis induced by phosphatase and tensin homologue (PTEN) deficiency [25, 26].

Although somatic mutations such as the translocation or loss of heterozygosity of CBP and p300 have been observed in leukaemia as well as in solid tumours such as hepatocellular carcinoma, breast cancer and CRC, genetic mutations in these two genes remain rare [27–33].

In an analysis of 222 cancer samples, truncating mutations in p300 were only observed in six out of 107 (5.6%) cell lines and two out of 115 (1.7%) primary tumours [34–36]. CBP mutations are even rarer; only two heterozygous truncations and no further mutations were discovered in 63 cell lines. In 116 primary tumours, truncating mutations could not be detected [36]. These findings support our hypothesis regarding the roles of CBP and p300 as central chromatin modifiers and suggest that epigenetic therapies specifically targeting CBP or p300 may serve as a potential option for the treatment of a subset of colorectal tumours.

This potential targeting is further supported by our finding that approximately two-thirds of tumour cells from our patients highly expressed CBP and p300, stressing their importance in the development and progression of cancer as transcriptional coactivators and HATs.

At least in our hands, stronger expression of CBP, but not p300, seems to increase CSS. Furthermore, our results revealed CBP as an independent prognostic factor regardless of tumour stage or localization, which could not be shown for p300. Cooperation between CBP and p300 was not verifiable in this cohort of patients, supporting current evidence that the two HATs play different roles in tumourigenesis. This hypothesis is strengthened by in vitro and in vivo analyses that showed different specificities and selectivities for CBP and p300 in the acetylation of histones, the inability of CBP to rescue the growth of p300-deficient carcinoma cell lines and an inverse prognostic correlation in CRC [24, 37, 38].

Our results may introduce CBP as a potential target in a subset of colorectal cancer patients. Our findings are further supported by an analysis by Du et al. [39]*.* Their results demonstrated global histone deacetylation in CRC cell lines caused by 5-fluorouracil (5-FU), which is the standard chemotherapeutic agent in colorectal cancer. Additionally, they showed that 5-FU was capable of reducing the ability of CBP and p300 to bind to chromatin and thereby inducing their degradation. Interestingly, blocking CBP and p300 degradation resulted in an enhancement in 5-FU’s cytotoxicity to CRC cells, indicating that the degradation of CBP and p300 is relevant to cellular resistance to 5-FU. By analysing 262 samples from colorectal cancer patients receiving 5-FU treatment via immunohistochemistry, Du et al. showed that high expression of CBP and p300 significantly correlated with prolonged disease-free survival (DFS) and decreased early progression. Taken together, CBP and p300 might represent not only prognostic biomarkers but also predictive biomarkers of chemo-sensitivity to 5-FU treatment, thereby distinguishing responders from non-responders to stratify patients for CRC therapy.

Taken together, the prognostic capacities of CBP and p300 have been investigated in previous studies with partially controversial results [24, 39–41]. Both of these highly homologous transcriptional coactivators are essential in apoptosis, cell transformation, differentiation and growth, as well as in CRC [42]. CBP and p300 both acetylate a variety of transcription-regulating proteins, including oncogenes and tumour suppressors such as p53 [43–45]. In recent years, efforts have been made to target CBP and p300, including designing small-molecule inhibitors with heterogeneous efficacy [46–48]. However, our findings may provide better insight into the clinical significance of CBP and p300 in patients suffering from CRC, although the distinct roles of these two HATs in CRC remain incompletely understood.

## Conclusions

In this patient cohort, high expression of CBP was correlated with improved long-term outcomes. This histone acetyltransferase could therefore represent a potential biomarker for stratifying therapeutic regimens for patients suffering from colorectal cancer. Inhibitors of CBP have already been implemented in preclinical trials. It is desirable to find not only prognostic biomarkers but also particularly predictive biomarkers to predict the success of therapies and to prevent severe side effects of therapies from which not every patient benefits. At least in our hands, CBP may represent both.

As genetic mutations in these two genes are known to be rare, we and others postulate central epigenetic functions for these two proteins in tumour initiation and progression, and both enzymes may therefore be feasible targets for anticancer treatment.

The small number of patients and the fact that immunohistochemistry detects only expression, not activity, represent the limitations of our study.

Further studies on CBP and p300 are desirable to evaluate the future potential of these two proteins in cancer therapy. Our findings should be further evaluated and verified in upcoming clinical trials.

## Data Availability

All data generated or analysed during this study are included in the published article.

## Abbreviations

5-FU: 5-Fluorouracil
ARR: Anterior rectal resection
CBP: CREB-binding protein
CRC: Colorectal cancer
CSS: Cancer-specific survival
DNA: Deoxyribonucleic acid
FFPE: Formalin-fixed, paraffin-embedded
HAT: Histone acetyltransferase
HDAC: Histone deacetylase
LARR: Lower anterior rectal resection
OS: Overall survival
PME: Partial mesorectal excision
PTEN: Phosphatase and tensin homologue
UICC: Union International Contre le Cancer
UMG: University Medical Center Göttingen
